# Modic Changes in Patients Who Have Undergone Anterior Cervical Discectomy and Fusion: The Correlation With Fusion Success and Subsidence

**DOI:** 10.1111/os.14377

**Published:** 2025-02-24

**Authors:** Yifei Deng, Xiang Zhang, Xiaqing Sheng, Beiyu Wang, Ying Hong, Xin Rong, Chen Ding, Jingjing An, Hao Liu

**Affiliations:** ^1^ Department of Orthopaedic Surgery West China Hospital, Sichuan University Chengdu China; ^2^ Operating Room, Department of Anesthesiology West China Hospital, Sichuan University Chengdu China

**Keywords:** anterior cervical discectomy and fusion (ACDF), fusion, modic change (MC), neck pain, subsidence

## Abstract

**Objective:**

There is a lack of research on modic change (MC) in the cervical spine, especially regarding its impact on patients following anterior cervical discectomy and fusion (ACDF). Some researchers strongly believe that MC may affect the prognosis after anterior cervical surgery. Thus, this study aimed to assess MC in patients who underwent ACDF, investigating its incidence, risk factors, and correlation with fusion success and subsidence rate.

**Methods:**

We retrospectively reviewed 154 patients who underwent single‐level ACDF from January 2010 to December 2020, with a minimum follow‐up of 12 months. Preoperative and postoperative clinical and radiological parameters were recorded at pre‐operation, 1 week, 3 months after operation, and the last follow‐up. The primary outcomes were the fusion rate and cage subsidence. Radiological measurements, including overall (Cobb C) and segmental cervical angle (Cobb S), anterior disc height (ADH), and posterior disc height (PDH) were also recorded. The independent *t*‐test or Mann‐Whiteny *U* test was used to compare continuous data, and categorical variables were assessed using the Pearson's chi‐square test of Fisher's exact test. Logistic regression analysis was also adopted to distinguish corresponding factors related with the progress of MC.

**Results:**

Of the 154 patients, the incidence of MC was 44.2% (68/154). The group with MC showed a larger proportion of males and osteoporosis. The fusion rate of those with MC was 88.2% (60/68) while that in the non‐MC group was 97.7% (84/86, *p* = 0.02). The MC group presented a subsidence rate of 27.9%, which was substantially higher than in the non‐MC group (9.3%, *p* < 0.01). NDI and VAS neck was significantly higher in the MC group than in the non‐MC group (*p* = 0.014; *p* = 0.039). Sex and osteoporosis were distinguished as independent factors related to MC by regression analysis (*p* = 0.006; *p* = 0.026).

**Conclusion:**

Preoperative MC could adversely hinder the fusion process and may increase the incidence of subsidence, affecting clinical outcomes of those underwent ACDF. Patients with MC, especially type 1 MC, are more easily suffered from neck pain than those without MC. Male sex and osteoporosis were risk factors for MC. In order to achieve a better bony fusion and avoid cage subsidence in those with MC, we encourage patients to prolong their immobilization duration with a cervical collar and precisely manage osteoporosis during the peri‐operative period.

## Graphical abstract

Patients with MC after ACDF are suffered from subsidence at different follow‐up time. Through our observation, we found that subsidence rate in the MC group (27.9%) was also significantly higher than that in the non‐MC group (9.3%).

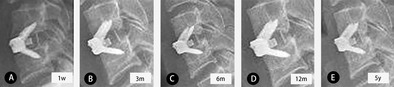

## 1 Introduction

Modic change (MC), first witnessed and defined by Modic et al., was the signal change at the subchondral vertebral endplate [1]. The pathology of MC concluded inflammation, edema, and sclerosis, and thus could be divided into three types [2]. MC have been reported to intertransmit with each types [3, 4]. In the past, MC have been extensively reported and studied in the lumbar spine [5, 6]. Research have barely concentrated on MC in the cervical spine, let alone its effect on prognosis of ACDF. The reported incidence of MC ranges from 3% to 40.4% in the cervical spine [7, 8, 9, 10]. Among all these studies concerning about MC, some have regarded MC as being related to neck pain and axial symptoms. In contrast, others hold that MC have nothing to do with clinical symptoms [11, 12]. Moreover, when it comes to the effects of MC on those following ACDF, the results seem more ambiguous.

When treating patients suffered from degenerative disc disease (CDDD), anterior cervical discectomy and fusion (ACDF) is the common procedure that most surgeon would choose [13, 14]. However, complications like adjacent segment degeneration (ASD) have been increasingly noticed in those who underwent ACDF, and the incidence is mounting with a longer follow‐up [15]. Besides, researchers have reported that MC may lead to the post‐operative complications of ACDF. For instance, Li et al. have reported that MC at the cranial or caudal operative level may lead to a higher incidence of ASD following ACDF [16]. Yang et al. have found that the presence of MC was the leading cause of persisting symptoms after thorough depression of ACDF [17]. Huang et al. persisted that MC may affect the early fusion rate of ACDF [12].

Non‐fusion and subsidence remain a common complication after ACDF and may lead to persistent post‐operative pain. Some of them may even suffer from a secondary surgery. According to some previous researchers, the pathology of MC was closely related with osteoclast‐related cytokines. Activated osteoclast may delay the fusion processes and result subsidence after ACDF. Thus, the purpose of this study was to: (i) explore MC's prevalence and radiological characteristics; (ii) illustrate the effect of MC on fusion and subsidence; (iii) their effect on clinical prognosis; (iv) evaluate factors associated with MC.

## 2 Materials and Methods

### 2.1 Study Population

The current study (Ethics Committee on Biomedical Research, No. 2020–684) has acquired approve of the institutional review board. We retrospectively reviewed 154 patients with single‐segment ACDF using Zero‐Profile (Zero P; Synthes GmbH, Oberdorf, Switzerland) from 2010 to 2020, with a minimum follow‐up of 12 months.

The inclusion criteria were as follows: (i) single‐segment CDDD who underwent single‐segment ACDF; (ii) presenting with persisting radiculopathy or myelopathy; (iii) not response to conservative management for at least 3 months. The exclusion criteria were: (i) prior surgery operated on the C3‐7 cervical spine; cervical deformity; (ii) others: Including carcinoma, fracture, infection, rheumatoid arthritis, as well as metabolic bone disease; (iii) multilevel or hybrid surgery.

### 2.2 Definition of MC

**FIGURE 1:**
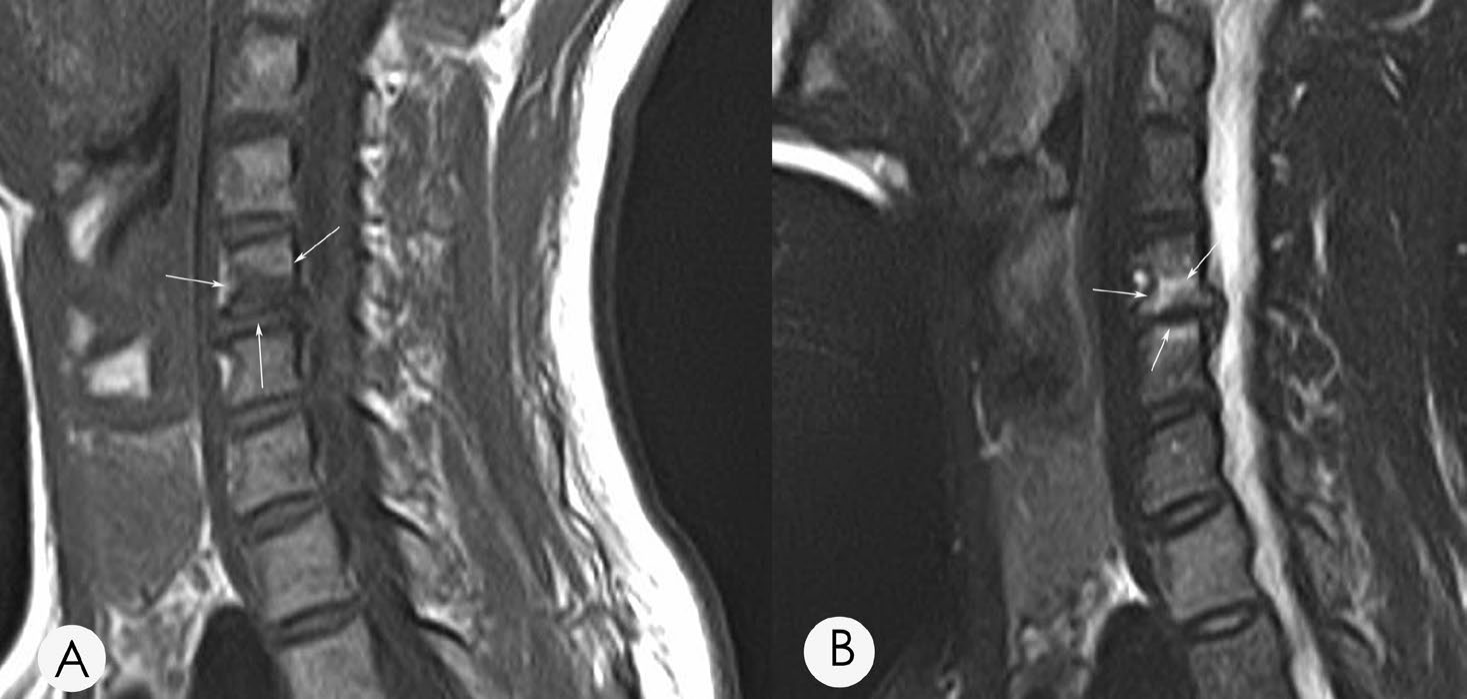
(A) A T1‐weighted magnetic resonance imaging (MRI) of a 54‐year‐old female demonstrates hypointensity at the inferior endplate of C5. (B) A T2‐weighted MRI of the same patients demonstrates hyperintensity, suggesting a type 1 MC. Arrows indicate the location of the identified MC lesion on T1 and T2 imaging.

**FIGURE 2:**
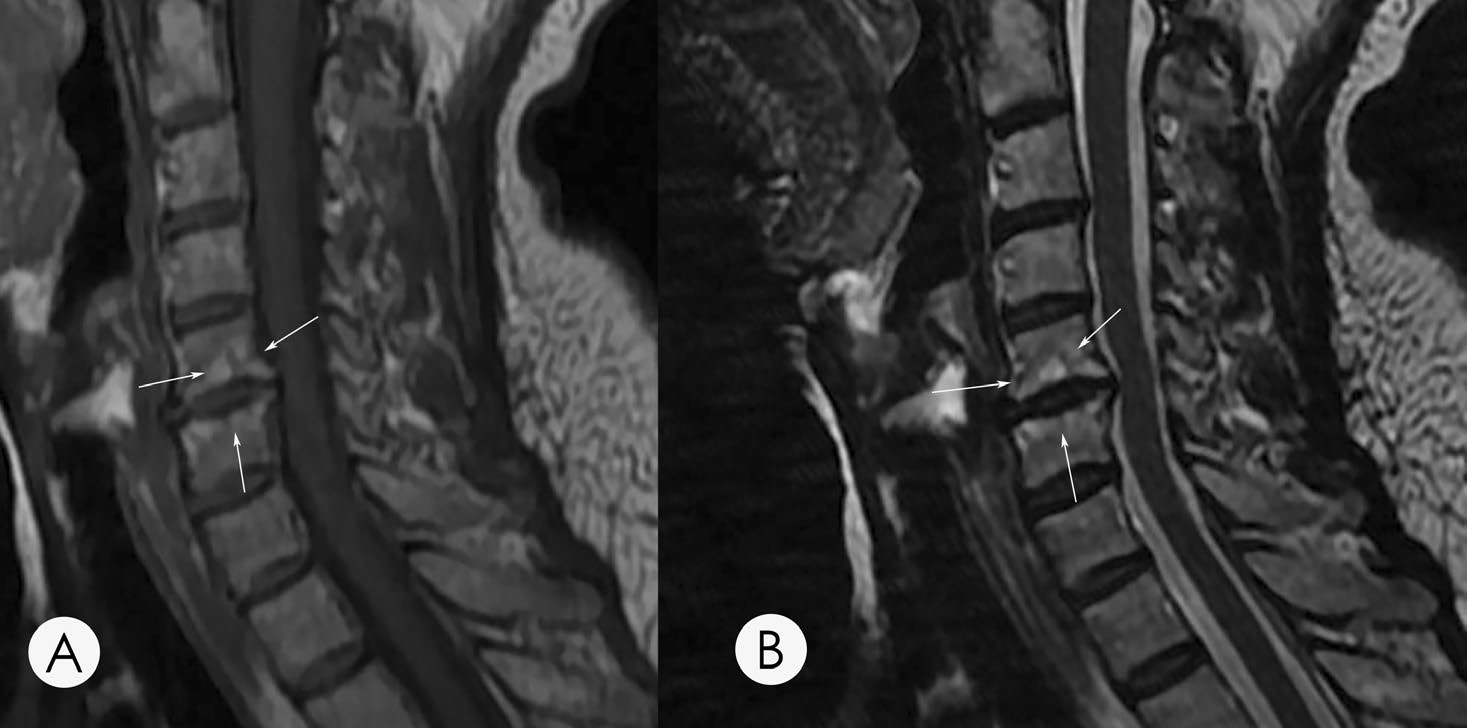
(A) A T1‐weighted magnetic resonance imaging (MRI) of a 48‐year‐old female shows hyperintensity at the inferior endplate of C4 and superior endplate of C5. (B) A T2‐weighted MRI of the same patients demonstrates hyperintensity, suggesting a type 2 MC. Arrows indicate the location of the identified MC lesion on T1 and T2 imaging.

**FIGURE 3:**
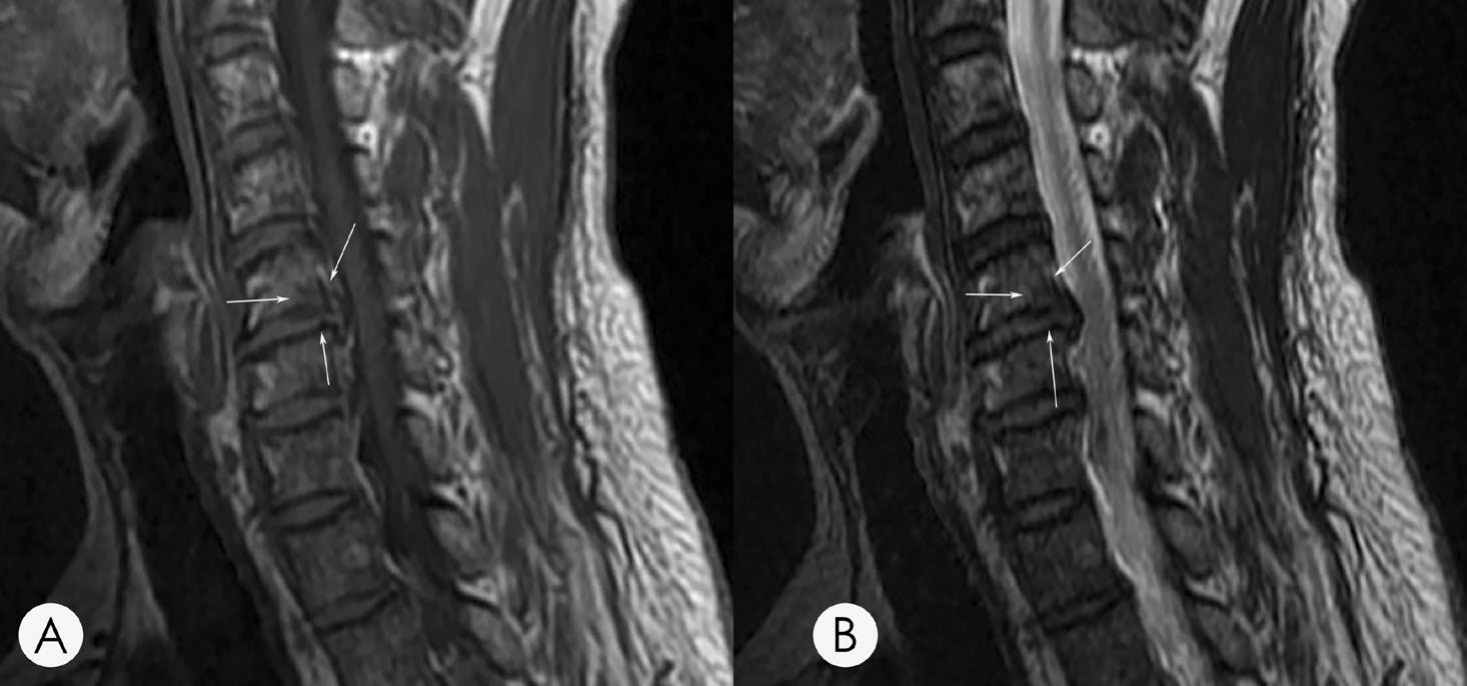
(A) A T1‐weighted magnetic resonance imaging (MRI) of a 51‐year‐old male demonstrates hypointensity at the inferior endplate of C5. (B) A T2‐weighted MRI of the same patient demonstrates hypointensity, suggesting a type 3 MC. Arrows indicate the location of the identified MC lesion on T1 and T2 imaging.

Based on the characteristic on MRI, MC were typically divided into three different groups. Type 1 MC is characterized by a low signal on T1‐weighted and a high signal on T2‐weighted sequences (Figure 1). Type 2 MC illustrates a high signal on both T1‐weighted and T2‐weighted sequences (Figure 2). Type 3 MC exhibits a low signal on both T1‐ and T2‐weighted sequences (Figure 3).

### 2.3 Surgical Technique

A classical Smith‐Robinson way was adopted and conducted by the same professor. After targeted at the operated segment, an incision was made transversely. According to our experience, both the anterior and posterior longitudinal ligament were regularly removed. Then, the intervertebral disc was cut as much as we can to acquire a thorough decompression. Endplate preparation combined with a high‐speed burr was another indispensable procedure. When all the steps were finished, we selected an appropriate Zero‐P with the help of implant trial. Intraoperative X‐ray were recorded to make sure that the Zero‐P was positioned correctly. Last but not least, saline was irrigated to remove excessive tissues and bony dust in every single case. All patients were instructed to immobilize with a soft cervical collar for at least 3 months.

### 2.4 Evaluation of Clinical and Radiological Outcomes

Japanese Orthopedic Association (JOA) score, Neck Disability Index (NDI) score, and visual analog scale (VAS) arm and neck score were used to assess clinical outcomes. Radiographs of each patient were also collected at each follow‐up time.

Radiological measurements included cervical lordosis (CL) and functional segmental unit angle (FSUA), anterior disc height (ADH), and posterior disc height (PDH). C2‐7 Cobb angle was applied to define CL. FSUA was the angle between the lines from the cranial vertebrae's superior endplate and the caudal vertebrae's inferior endplate at the surgical level. ADH and PDH were measured at intervertebral disc height. Once the average scores of ADH and PDH decreased more than 2 mm, we defined as subsidence. Radiographs including X‐ray and CT were recorded to assess the fusion process. Bony fusion was achieved as long as one of the following signs was observed: Angular motion on dynamic X‐ray was less than 2°; a radiolucent gap between the implant and endplates was disappear; continuous trabeculae bone presented at the implant‐endplate interface.

We compared the differences in radiological parameters like CL, FSUA, ADH, PDH, subsidence, and fusion between patients with and without MC. Clinical outcomes concluded JOA, NDI and VAS neck/arm. Three spine surgeons with 10 years of expertise in cervical spine surgery independently evaluated all the radiological and clinical parameters at pre‐operative, post‐operative, 3‐month and at last follow‐up, and the average score of corresponding data was acquired for further analysis [18].

### 2.5 Statistical Analysis

SPSS 25.0 software (SPSS Inc. Chicago, IL, USA) was utilized for statistical analyses. The intraclass correlation coefficient (ICC) was applied to evaluate the interobserver reliability of the radiological parameters. For continuous variables, data were recorded as mean ± standard deviation. To compare the difference between the groups with and without MC, independent *t*‐test (normal distribution) or Mann–Whitney *U* test (abnormal distribution) was applied for continuous variables while Pearson Chi‐Square or Fisher's Exact Test was conducted for categorical variables. *p* value less than 0.05 was defined as statistically significant. Last but not least, risk factors that were significantly associated with MC were distinguished by logistic regression analysis.

## 3 Results

### 3.1 Patient Demographics

**FIGURE 4:**
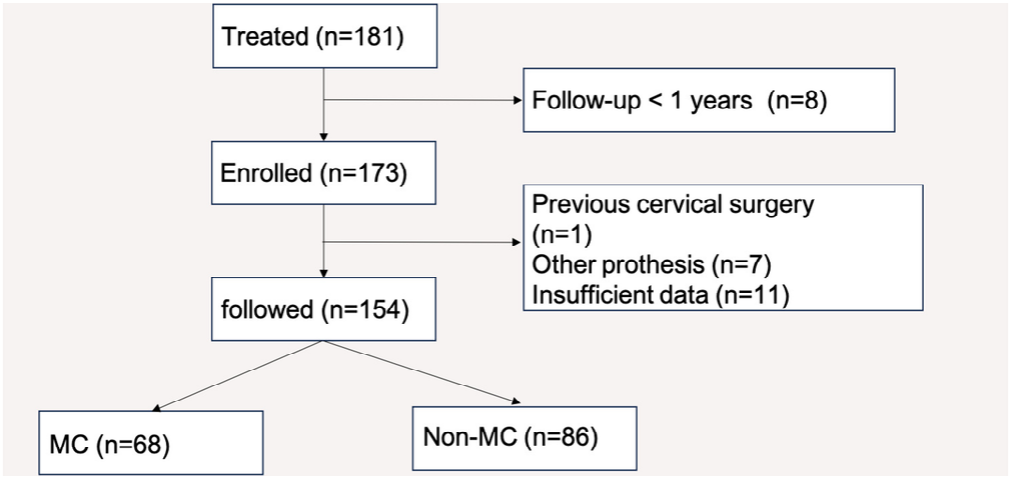
Flowchart of patient enrollment.

There were altogether 154 patients enrolled in our study from 181 patients receiving ACDF from 2010 to 2020. Among the 27 excluded patients, eight did not achieve 1 year follow‐up, one had previous cervical surgery, seven had other prothesis, 11 had an insufficient data (Figure 4). MC was witnessed in 68 patients and defined as MC group while 86 patients were divided to the non‐MC group. The average age was 52.14 ± 11.32 years old. The mean follow‐up time was 22.22 ± 14.13 months. Not only age, sex, follow‐up time, operation time, blood loss, level distribution, but also BMI, smoking, osteoporosis, hypertension, diabetes were recorded. Table 1 displays the detailed data of patient characteristic. Complications like graft malposition or screw loosening were recorded. No patients were reported to conduct another cervical spine surgery on the operated level.

**TABLE 1:** Patient's demographic characteristic.

Variable	Value (%)
Number of patients	154
Gender (M/F)	80/74
Age (years)	52.14 ± 11.32
Follow‐up (months)	22.22 ± 14.13
BMI	25.20 ± 4.25
Smoking	37 (24%)
Osteoporosis	15 (9.7%)
Hypertension	32 (20.8%)
Diabetes	16 (10.4%)
Operation time	97.85 ± 17.34
Blood loss	51.10 ± 15.87
Level distribution	
C3/4	16 (10.4%)
C4/5	14 (9.1%)
C5/6	114 (74.0%)
C6/7	10 (6.5%)
Modic change	68 (44.2%)
Type 1	6 (3.9%)
Type 2	38 (24.7%)
Type 3	24 (15.6%)

### 3.2 Prevalence of MC

The ICC for radiological measurements exemplified great agreement (0.89 for CL, 0.93 for FSUA, 0.91 for ADH, and 0.95 for PDH, respectively). A total of 154 objectives were enrolled in this study, MC was witnessed in 68 patients (44.2%). Among those with MC, six patients were diagnosed with type 1 MC; 38 patients were defined as type 2 MC, and 24 patients were assigned to type 3 MC. The MC group exhibited a significantly higher proportion of male sex and osteoporosis compared to the non‐MC group. Variables, such as age, BMI, follow‐up period, and operated levels, exemplified no significant differences between the groups with and without MC (Table 2).

**TABLE 2:** The fusion/subsidence rate and univariate analysis that may be related to MC.

Variable	MC	Non‐MC	*t*/*x* ^2^/*z*	*p*
No. of patients, *n*	68	86	—	—
Gender (M/F)	44/24	36/50	7.940	< 0.01
Age, year	51.29 ± 9.28	52.81 ± 12.73	−0.826	0.41
Follow‐up, months	20.88 ± 11.50	23.28 ± 12.89	−1.085	0.28
Blood loss	51.76 ± 16.39	50.58 ± 15.51	0.458	0.65
Operation time	99.64 ± 17.50	96.45 ± 17.18	1.131	0.26
BMI	25.41 ± 3.93	25.03 ± 4.50	0.545	0.59
Smoking	17	20	0.063	0.80
Osteoporosis	13	6	5.175	0.02
Hypertension	15	17	0.121	0.73
Diabetes	8	8	0.247	0.62
Fusion rate	60 (88.2%)	84 (97.7%)	5.572	0.02
Subsidence	19 (27.9%)	8 (9.3%)	9.124	< 0.01

Abbreviation: MC, Modic change.

The majority of the patients relieved from symptoms after cervical spine surgery, accompanying with increased JOA and decreased VAS and NDI scores. NDI in the MC group was significantly higher than that in the non‐MC group at pre‐operative, post‐operative and last follow‐up time. VAS neck in the MC group was significantly higher than that in the non‐MC group at post‐operative and last follow‐up time. There was no significant difference of JOA and VAS arm between those with and without MC at any follow‐up period (Table 4). Furthermore, a subgroup analysis was conducted and revealed that NDI and VAS neck in the type 1 MC group was significantly higher than that in the type 2 and type 3 MC group (Table 5). In a word, patients with MC, especially type 1 MC, are more easily suffered from neck pain compared with those without MC.

### 3.3 Cervical Radiological Parameters

**FIGURE 5:**
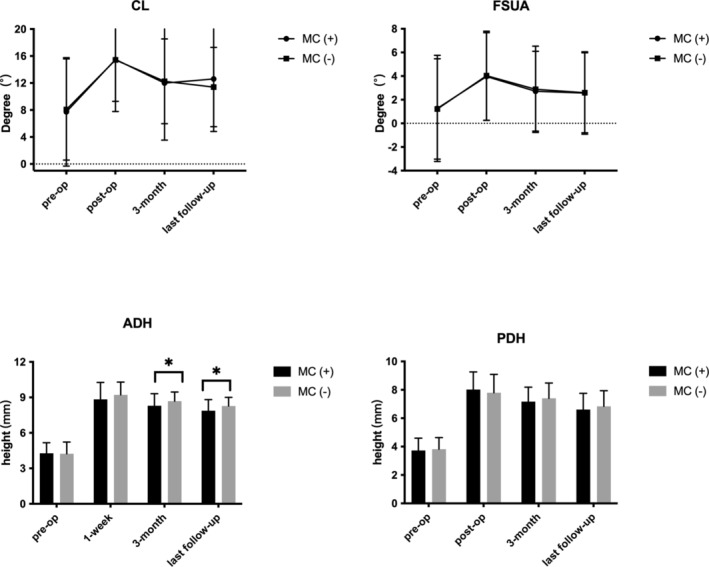
Comparison of CL, FSUA, ADH, and PDH in MC group and non‐MC group. Results are expressed as mean ± SD. A dragger represents a significant difference between the two groups. ADH in the MC group was significantly lower than in the non‐MC group at 3 months and the last follow‐up.

As demonstrated in Table 3, cervical sagittal measurements including CL and FSUA in MC and non‐MC group exerted no significant difference at any follow‐up time. For patients in the MC group, the ADH at 3‐month follow‐up (8.30 ± 1.02 vs. 8.69 ± 0.77, *p* = 0.01) and last follow‐up (7.87 ± 0.94 vs. 8.28 ± 0.73, *p* = 0.004) was significantly lower compared with that in the non‐MC group. In contrast, PDH in both groups showed no significant difference at any follow‐up time (Figure 5).

**TABLE 3:** Cervical radiological parameters of patients with MC or without MC.

Variables	MC (+)	MC (−)	*t*/*x* ^2^/*z*	*p*
CL				
Pre‐operation	7.72 ± 1.57	8.08 ± 1.78	−0.285	0.776
1 week	15.49 ± 3.01	15.42 ± 3.04	0.071	0.943
3 months	12.00 ± 1.75	12.25 ± 1.70	−0.209	0.835
Last follow‐up	12.61 ± 1.85	11.40 ± 1.86	1.070	0.286
FSUA				
Pre‐operation	1.26 ± 0.63	1.22 ± 0.75	0.063	0.950
1 week	3.96 ± 0.96	4.03 ± 0.93	−0.108	0.914
3 months	2.72 ± 1.37	2.88 ± 1.64	−0.285	0.776
Last follow‐up	2.57 ± 1.48	2.59 ± 1.39	−0.037	0.870
ADH				
Pre‐operation	4.28 ± 0.89	4.23 ± 0.99	0.293	0.770
1 week	8.83 ± 1.43	9.21 ± 1.08	−1.816	0.071
3 months	8.30 ± 1.02	8.69 ± 0.77	−2.620	0.010
Last follow‐up	7.87 ± 0.94	8.28 ± 0.73	−2.915	0.004
PDH				
Pre‐operation	3.73 ± 0.87	3.82 ± 0.82	−0.654	0.514
1 week	7.79 ± 1.29	8.01 ± 1.25	−1.061	0.290
3 months	7.12 ± 1.08	7.43 ± 1.02	−1.363	0.175
Last follow‐up	6.61 ± 1.14	6.83 ± 1.10	−1.218	0.225

Abbreviations: ADH, anterior disc height; CL, cervical alignment; FSUA, functional segmental unit angle; MC, modic change; PDH, posterior disc height.

**TABLE 4:** The JOA, NDI, and VAS scores of patients with or without MC.

	MC (+)	MC (−)	t/x^2^/z	*p*
Pre‐JOA	9.03 ± 2.47	8.90 ± 2.52	0.308	0.758
Po‐JOA	12.32 ± 2.13	12.62 ± 2.08	−0.862	0.390
Last‐ JOA	15.35 ± 1.56	15.05 ± 1.78	1.114	0.267
Pre‐NDI	33.90 ± 3.83	32.47 ± 2.87	2.244	0.027
Po‐NDI	20.67 ± 3.72	18.87 ± 3.41	2.638	0.010
Last‐ NDI	9.04 ± 2.77	7.95 ± 1.73	2.501	0.014
Pre‐VAS neck	7.78 ± 1.85	7.38 ± 1.82	1.329	0.186
Po‐VAS neck	4.12 ± 2.07	3.43 ± 1.48	2.404	0.017
Last‐VAS neck	1.82 ± 1.28	1.47 ± 0.85	2.081	0.039
Pre‐VAS arm	7.68 ± 1.52	7.29 ± 1.58	1.531	0.128
Po‐Vas arm	3.50 ± 1.52	3.14 ± 1.38	1.537	0.126
Last‐VAS arm	2.44 ± 1.14	2.27 ± 1.13	0.944	0.347

Abbreviations: JOA, Japanese Orthopedic Association; MC, modic change; NDI, neck disability index; VAS, visual analog scale.

**TABLE 5:** The JOA, NDI, and VAS scores of patients with different type of MC.

	Type 1 MC	Type 2 MC	Type 3 MC	*t*/*x* ^2^/*z*	*p*
Pre‐JOA	9.00 ± 1.55	9.32 ± 2.59	8.58 ± 2.41	0.651	0.525
Po‐JOA	11.67 ± 2.73	12.27 ± 1.84	12.42 ± 2.45	0.311	0.734
Last‐JOA	14.33 ± 1.52	15.42 ± 1.55	15.50 ± 1.69	1.439	0.245
Pre‐NDI	37.67 ± 2.75	33.60 ± 3.83	33.06 ± 3.71	3.748	0.031
Po‐NDI	26.83 ± 2.72	20.17 ± 3.13	19.31 ± 3.07	14.741	< 0.01
Last‐NDI	13.33 ± 1.75	8.60 ± 2.28	8.25 ± 2.56	11.662	< 0.01
Pre‐VAS neck	8.83 ± 1.41	7.92 ± 1.81	7.29 ± 2.03	1.971	0.147
Po‐VAS neck	7.00 ± 2.10	4.00 ± 2.58	3.58 ± 2.39	8.092	0.001
Last‐VAS neck	3.83 ± 1.56	1.74 ± 1.08	1.46 ± 0.51	10.966	< 0.01
Pre‐VAS arm	8.33 ± 1.37	7.79 ± 1.38	7.17 ± 1.76	1.287	0.150
Po‐Vas arm	3.33 ± 1.35	3.79 ± 1.34	3.08 ± 1.77	1.657	0.199
Last‐ VAS arm	2.67 ± 1.03	2.52 ± 1.01	2.21 ± 1.14	0.798	0.493

Abbreviations: JOA, Japanese Orthopedic Association; MC, modic change; NDI, neck disability index; VAS, visual analog scale.

**TABLE 6:** Risk factors analysis for MC.

Risk factors	*B*	Wald	*P*	OR	95% CI
Gender	0.958	7.461	0.006	2.605	1.311–5.180
Age	−0.11	0.549	0.459	0.989	0.960–1.019
BMI	0.034	0.661	0.416	1.035	0.953–1.125
Smoking	−0.10	0.001	0.992	0.990	0.147–6.670
Hypertension	−0.150	0.018	0.894	0.861	0.096–7.734
Osteoporosis	1.878	4.933	0.026	6.543	1.247–34.34
Diabetes	−0.653	0.447	0.504	0.520	0.077–3.543

Abbreviations: BMI, body mass index; MC, modic change.

Concerning about bony fusion, the fusion rate was 88.2% in the MC group and 97.7% in the non‐MC group respectively at the last follow‐up. Fusion rate between MC and non‐MC group was significantly different (*p* < 0.05). As for the subsidence, the incidence was 27.9% in the MC group and 9.3% in the non‐MC group, respectively. Such differences between the two groups were significantly different.

**FIGURE 6:**
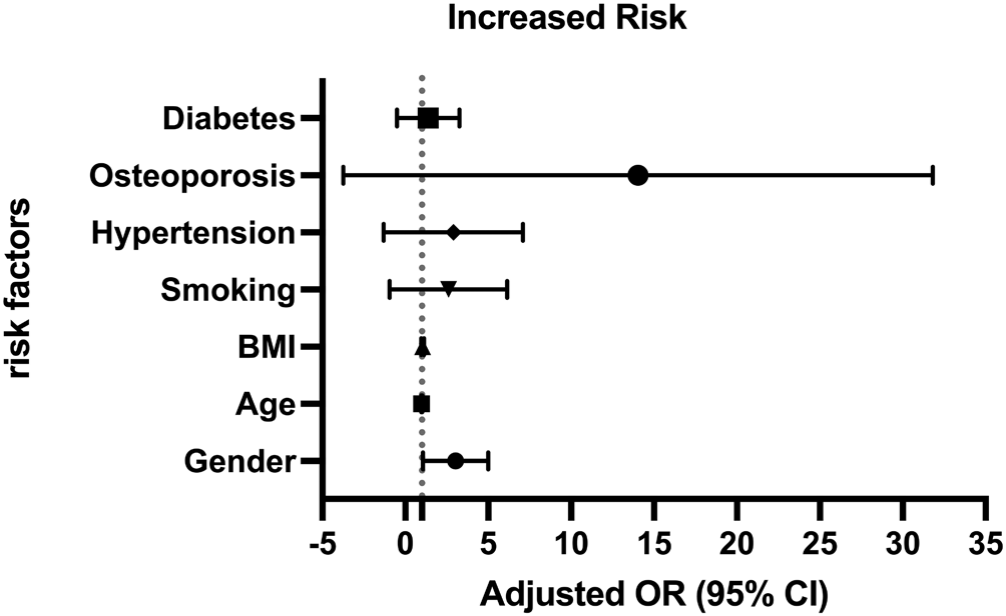
Multivariate logistic regression showed that male sex and osteoporosis were risk factors that were significantly associated with MC.

Male sex and osteoporosis were found as risk factors which were significantly related to MC according to logistic regression analysis (Figure 6; Table 6).

## 4 Discussion

### 4.1 Main Findings of This Study

As far as we concerned, the current study is the first one to observe the prevalence of MC in patients who underwent ACDF and to further examine the association between MC and fusion and implant subsidence. Besides, we figured out the risk factors concerning MC. The prevalence of MC in patients who underwent ACDF is 44.2%. Preoperative MC could adversely hinder the fusion process and may increase the incidence of subsidence, affecting clinical outcomes of those underwent ACDF. Patients with MC, especially type 1 MC, are more easily suffered from neck pain than those without MC. Logistic regression analysis revealed that male sex and osteoporosis were risk factors for MC.

### 4.2 Prevalence

The consequences of our research showed that the incidence of MC was higher at 44.2%. The prevalence of cervical MC varies a lot among existing researches. Peterson et al. reviewed 118 patients' cervical spine and recorded the incidence of MC was 16% [19]. Baker et al. enrolled 861 patients and reported 356 (41.3%) patients observed with MC [11]. Meanwhile, Li et al. have reported the incidence of MC was 58.5% [10]. The incidence of MC of our study was higher at 44.2%. Such prevalence was accord with the previous report. Discrepancies between these studies may be owing to differences in demographic data like age, and so on.

### 4.3 Correlation Between MC and Disc Height/Subsidence

Cervical radiographs showed that the MC group suffered a significant decrease in ADH at the 3‐month follow‐up and last follow‐up. However, no difference was noticed concerning PDH at any follow‐up time. Cage subsidence is common in patients after ACDF, and such complications are easily observed in zero‐profile anchored spacer [18]. A higher proportion of MC patients suffered from subsidence in the current study.

Interestingly, disc height loss at the anterior vertebrae's anterior part is where the MC mostly happened. Such difference between the two groups could be partly owing to the activated osteoclasts. According to some previous researchers, they observed that intervertebral discs near to MC may own significantly larger amounts of factors, like IL‐6, IL‐8, TNF [20], and other osteoclast‐related cytokines, such as RANKL, M‐CSF, NFATc1, RUNX1, OSCAR, than the discs without adjacent MC [21, 22]. These factors or cytokines may alter trabecular bone mass. A study explicitly targeting MC biopsy showed that the bone turnover rate of MC was high, and it is inferred that this is mainly due to the inflammatory process [22]. Typically, bone formation or resorption in those with MC is a temporary and transient pathology, counted mainly on the ratio of osteoblasts to osteoclasts. Teichtahl et al. discovered that MC were tightly bonded to a decrease in disc height, and the risk of MC nearly mounted up 1.6 times for every 1 mm loss in intervertebral height [23]. Li et al. have demonstrated a negative correlation between MC and disc height. Their observation concluded that MC may obstruct the delivery of nutritional materials transportation from vertebral vessels to the intervertebral disc, leading to metabolic disorder and resulting in degeneration and a decrease in disc height at last [24].

### 4.4 Correlation Between MC and Fusion

Through our observation, it is harder for patients with MC to achieve complete bony fusion than those without MC. Huang et al. persisted that MC may affect the early fusion rate of ACDF [12]. Researchers strongly believed that MC was closely related to the inflammatory process in vertebral endplates and subchondral bone marrow. Inflammatory cytokines could activate osteoclasts, and disrupted the ratio between osteoblasts and osteoclasts, and ultimately led to excessive bone resorption [25, 26, 27, 28]. This could explain why a more significant proportion of patients with MC did not achieve bony fusion at the last follow‐up. Xiao et al. also observed less patients in the MC group, especially those with sclerotic endplate, achieved completed bony fusion [29].

### 4.5 Clinical Outcomes, Pathology and Risk Factors for MC

Through the current observation, NDI and VAS neck was significantly higher in the MC group compared with non‐MC group, while JOA and VAS arm exerted no significant differences in the MC and non‐MC groups. The illustrated consequences highly indicate that MC, especially type 1 MC may be associated with neck pain or other specific clinical symptoms.

Typically, MC is the subchondral endplate signal change of the spine and have been noticed and defined by Modic et al. [1] MC could be divided into three groups based on its unique representation on MRI [2]. The pathology of type 1 MC was closely related to inflammation, which is thought to be closely related to spine pain and may result in the persistence of neck or lumbar pain after spine surgery. Type 2 MC represents the fatty filtration of vertebral endplate and marrow. As for type 3 MC, many researchers believe that type 3 MC is a similar pathology like endplate sclerosis. Such three types of MC are not separated but are interacted and could be transformed from one to another [30].

Meanwhile, a few research have reported that MC may play a role in the prognosis of patients who have underwent ACDF. For instance, Li et al. reported that MC at the nearby segment may lead to a higher incidence of ASD following ACDF [16]. Zhou et al. have found that the presence of MC was the leading cause of persisting symptoms after thorough depression of ACDF [31]. Previously, plenty of studies devoted to MC have unveiled that such pathology are closely related to clinical symptoms and play an independent role in causing neck and lower back pain [32, 33, 34]. However, a meta‐analysis exemplified that MC was not the only reason for persistent symptoms existed following spine operation [35].

Male sex and osteoporosis were identified as independent factors, which may affect the incidence and progress of MC through logistic regression analysis. Baker et al. have illustrated a larger proportion of male existed in the type 2 MC group compared to the non‐MC group [11]. Karchevsky et al. have also pointed out that male sex plays an independent role in developing MC [36]. However, the vast majority of studies have not discovered a strong correlation between sex and the mechanical and biological properties of the intervertebral disc [37].

Osteoporosis, featured as decreasing bone mass and density, is always accompanying with trabecular and cortical bone microarchitecture destruction. Overall, about 10.2 million people more than 50 years old suffered from osteoporosis, causing enormous economic burden. Osteoporosis is reported to result in endplate injury. Repeated friction and mechanical factors, combined with inflammatory cytokines, may also promote the development of MC.

Interestingly, the pathology of osteoporosis and MC is closely related to increasing activity of osteoclasts. Xiao et al. have revealed that osteoporosis was significantly associated with MC [29]. Bisphosphonates, which are golden standard treatment for those diagnosed with osteoporosis, have recently been proved that could be used to reduce low back pain and decrease in area and volume affected by MC [38, 39, 40]. Our study was highly accord with above mentioned research and exemplified that osteoporosis and MC was tightly related. Our findings suggest that we could manage those diagnosed with MC, especially accompanying with osteoporosis, to take some bisphosphonates during peri‐operative time.

### 4.6 Limitations and Strengths

The current study is the first one to observe the prevalence of MC in patients who underwent ACDF and to further examine the association between MC and fusion and implant subsidence. However, several limitations of present study are worthy of our consideration. First, the single‐center cohort, small ample size, combined with retrospective feature may result in a potential bias. Second, the follow‐up time was not long enough to observe some long‐term complications that happened after ACDF. Third, the diagnosis of MC was made mainly based on pre‐operative MRI; postoperative MRI data was not available in the current study owing to metal artifacts. Last, this study did not include CT data to evaluate endplate sclerosis. Further analysis with large scale, multicenter, long‐term follow‐up with CT and postoperative MRI data needs to be conducted to elucidate the significance and relationships between MC and fusion and subsidence.

## 5 Conclusion

Among the patients following single‐segment ACDF, the prevalence of MC was 44.2%. Preoperative MC can adversely hinder the fusion process and increase the incidence of subsidence rate. MC has nothing to do with cervical alignment. However, patients with MC, especially with type 1 MC are more easily suffered from neck pain. Male sex and osteoporosis were risk factors for MC. To achieve a better bony fusion and avoid cage subsidence in patients with MC, we encourage to prolong the immobilization support with a cervical collar and manage osteoporosis systematically in the peri‐operative period.

## Author Contributions

Y.D., H.L., and J.A. contributed to the design of the study. Y.D. wrote the manuscript and prepared figures with the help of X.Z., B.W., X.R., Y.H., C.D., J.A., X.Z., X.S., and Y.D. conducted the statistical analyses. The results were discussed with H.L., J.A., X.Z., and X.S. All authors reviewed the manuscript.

## Ethics Statement

This study approved by the Institutional Review Board of West China Hospital. Informed consent was obtained from all individual participants included in this study.

## Consent

Informed consent was obtained from all individual participants included in this study.

## Conflicts of Interest

The authors declare no conflicts of interest.

## Data Availability

Datasets are available from the corresponding author on reasonable request.
